# Proteomic Analysis to Elucidate the Antibacterial Action of Silver Ions Against Bovine Mastitis Pathogens

**DOI:** 10.1007/s12011-015-0510-5

**Published:** 2015-10-03

**Authors:** Seog Jin Kang, Yong Il Cho, Ki Hyun Kim, Eun Seok Cho

**Affiliations:** National Institute of Animal Science, Rural Development Administration, Cheonan, 331-801 Republic of Korea

**Keywords:** Bactericidal effect, Mastitis, Proteomics, Silver ion

## Abstract

Silver ions act as a powerful, broad-spectrum antimicrobial agent and are known to kill over 650 different kinds of pathogens. We investigated the protein expression pattern and identity after silver ion treatment in *Escherichia coli* and S*taphylococcus aureus*, which are primarily responsible for the majority of bovine mastitis cases using proteomics. Two-dimensional electrophoresis showed that silver ion treatment significantly reduced 5 spot’s density in *E. coli* and *S. aureus*, respectively. We identified 10 proteins (alkyl hydroperoxide reductase C22 subunit, phosphoglucomutase, fructose-1-phosphate kinase, putative carbamoyl transferase, alpha-galactosidase, carbamate kinase, ornithine transcarbamoylase, fumarate hydratase class II, alcohol dehydrogenase, and conserved hypothetical protein) by matrix-assisted laser desorption ionization time of flight (MALDI-TOF). These results demonstrated that silver ions have bactericidal effects through energy deprivation, inhibition of DNA replication, and accumulation of oxidants in bovine mastitis pathogens and suggested that silver ions can be applied for the treatment of bovine mastitis.

## Introduction

Increasing resistance of pathogenic bacteria to bactericides and antibiotics due to the extensive use of these reagents now presents a more significant challenge for curing animal diseases [1, 2]. Thus, the interest to identify alternative, safe, and cost-effective bactericidal materials to cure animal disease is growing. Silver has long been known to have strong inhibitory and bactericidal effects [3–5]. A silver ion solution is composed of submicroscopic, positively charged silver particles in an aqueous medium. Silver ions act as a powerful, broad-spectrum, antimicrobial agent and are known to kill over 650 different kinds of pathogens including bacteria, viruses, fungi, parasites, and molds [6–9]. Furthermore, silver ions are also extensively used for food preservation, decontamination, and disinfection of medical supplies [10]. A positive charge is the primary feature of silver particles, enabling them to aggressively attach to microbes and kill them [11–13]. Additionally, the naturally small size of silver ions permits them to pass through cell membranes [13–15].

Several studies have been conducted to explain the bactericidal effects of silver ions [2, 16, 17]. It is generally believed that heavy metals react with SH groups, which leads to the inactivation of the cellular proteins [16, 18] and inhibition of bacterial oxygen metabolism. Recent studies proposed that silver ions also react with the thiol group of enzymes to inactivate them [12, 19] and interact with DNA [15], resulting in marked enhancement of pyrimidine dimerization by photodynamic reactions and possible prevention of DNA replication [12, 17]. However, the mechanism underlying the antimicrobial effects of silver is still not fully understood. In particular, proteomic insights that may help elucidate the bactericidal mechanism of silver ions are still lacking. Furthermore, no study has been conducted to examine the bactericidal effects of silver ions against bacteria that cause bovine mastitis.

The objective of this study was to investigate the mechanisms underlying the bactericidal effects of silver ions against bovine mastitis pathogens such as *Escherichia coli* (*E. coli*) and *Staphylococcus aureus* (*S. aureus*). For this, we used a proteomic approach to investigate the molecular aspects of this activity against *E. coli* and *S. aureus*.

## Materials and Methods

Two reference strains (*E. coli* O55 and *S. aureus* 305) were obtained from Animal Plant and Fisheries Quarantine and Inspection Agency (Anyang, Korea). The bacteria were sub-cultured using Muller-Hinton agar plates (BD, USA). A large amount of microorganisms was scraped from the agar plate and used to inoculate test tubes containing Muller-Hinton broth (MHB; BD, USA). The MHB cultures were incubated at 37 °C in ambient air until an optical density approximately equivalent to 1.0 McFarland standard (3 × 10^8^ CFU/mL) was achieved.

Silver ions (1000 μg/mL) were prepared from aqueous 0.01 M silver nitrate and various stabilizers (polyvinyl pyrrolidone and polyethylene) [20]. The pH of the solution was adjusted to 7.0 by adding sodium acetate. Silver ions were diluted (50 μg/mL) with pyrogen-free distilled water (Jungwei Pharma, South Korea).

### Sample preparation

*E. coli* and *S. aureus* (2 × 10^7^ cells) were incubated in MHB at 37 °C for 2 h with or without silver ion (50 μg/mL) and then centrifuged at 3000×*g* for 10 min. Cell pellets were washed twice with ice-cold PBS and sonicated for 10 s using a Sonoplus (Bandelin Electronic, Germany) in ice-cold lysis buffer (8 M urea, 4 % CHAPS, and 20 mM dithiothreitol). The lysates were centrifuged at 15,000×*g* for 10 min in a cold room, and the supernatants were collected.

### Two-dimensional protein gel electrophoresis

Protein samples (60 μg proteins) were subjected to isoelectrofocusing (IEF) on IPG strips (13 cm, pH 4–7) using the IPGphor IEF system(Ettan, USA). The first-dimension IEF conditions were 30 V for 10 h (rehydration) and 3500 V for 3 h with a total of 56 kVh. After IEF, the strips were incubated for 15 min in equilibration buffer (6 M urea, 30 % glycerol, 2 % SDS, and 50 mM Tris-HCl, pH 6.8) containing 1 % dithiothreitol and then for another 15 min in equilibration buffer containing 2.5 % iodoacetamide. The strips were transferred onto 12.5 % polyacrylamide slab gels containing 0.1 % SDS and electrophoresed. All gels were visualized by silver staining as described by Oakley et al. [21].

### Protein spot analysis

Quantitative analysis of digitized images of the gels was carried out using PDQuest software (version 7.0, BioRad, USA) according to the protocols provided by the manufacturer. Each spot was normalized by the total valid spot intensity. Proteins with expression levels that changed more than twofold compared to the control sample were selected for further analysis.

### MALDI-TOF MS/MS analysis

Gel slices were destained with a solution of 50 mM sodium thiosulfate and 15 mM potassium ferricyanide, washed with water, and dehydrated in acetonitrile. The dehydrated gel slices were subjected to overnight in-gel tryptic digestion using sequencing grade trypsin (Promega, USA) according to manufacturer’s instructions. The digested proteins were mixed with a saturation solution of R-cyano-4-hydroxycinnamic acid in 50 % acetonitrile and 0.1 % trifluoroacetic acid and spotted on the MALDI target plate. Peptide analysis was performed using matrix-assisted laser desorption ionization time of flight (MALDI-TOF) (Amersham Biosciences, UK). Peptides were evaporated with a N2 laser at 337 nm, and a delayed extraction approach was used. The peptides were then accelerated with a 20 kV injection pulse for time-of-flight analysis. Each spectrum was the cumulative average of 300 laser shots. The search program ProFound, developed by Rockefeller University (http://129.85.19.192/profound_bin/WebProFound.exe), was used for protein identification by peptide mass fingerprinting. Spectra were calibrated with trypsin auto-digestion ion peak *m*/*z* (842.510, 2211.1046) as an internal standard.

## Results

**Fig. 1:**
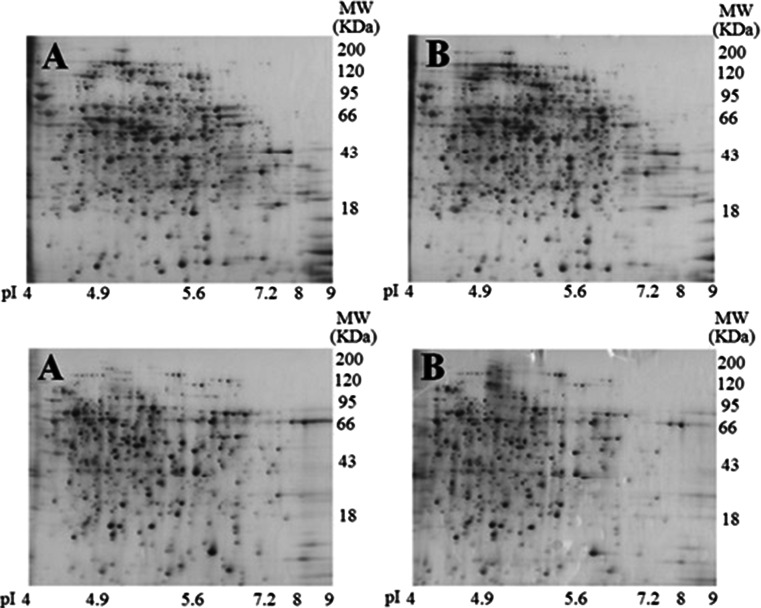
2-DE images from *E. coli* cell (*upper*) and *S. aureus* cells (*bottom*) treated with or without silver ions. Analytical alkaline silver-stained 2-DE protein patterns obtained from suspensions of *E. coli* and *S. aureus* after incubating the cells with 0 and 50 μg/mL solution for 2 h. 2-DE gel images of *S. aureus* cells treated with silver ions (**b**) and the untreated control (**a**)

No significant or clear changes were observed between the bacteria cells treated with or without silver ions in the two-dimensional protein gel electrophoresis (2-DE) images (Fig. 1). However, some spots in the 2-DE gels indicated decreased protein expression in silver ion-treated *E. coli* and *S. aureus* samples compared to the untreated cells. Spots corresponding to proteins with decreased expression caused by silver ion treatment were cut from the gels and analyzed using MALDI-TOF mass spectrometry (MS).

**Fig. 2:**
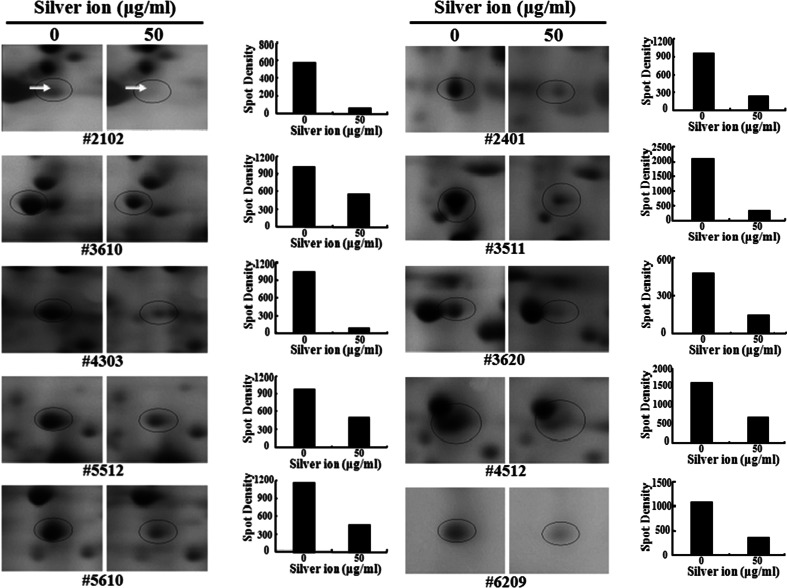
Comparison of the expression profiles of proteins corresponding to five spots from the control and silver ion-treated *E. coli* cells (*left*) and *S. aureus* (*right*). *Arrows* show the relative spot density changes in the control and silver ion-treated *E. coli* cells and *S. aureus*. *Bars* represent the average density based on three different observations

We investigated the results and revealed decreased expression of at least five proteins in each *E. coli* and *S. aureus* samples treated with silver ions (Fig. 2). Using the NCBI and Swiss-Prot databases for peptide mass fingerprinting, five proteins with decreased expression, the alkyl hydroperoxide reductase C22 subunit, phosphoglucomutase, fructose-1-phosphate kinase, putative carbamoyl transferase, and alpha-galactosidase, were identified in *E. coli* treated with silver ions (Table 1). The same fingerprinting databases identified the identity of five proteins with decreased expression, carbamate kinase, ornithine transcarbamoylase, fumarate hydratase class II, alcohol dehydrogenase, and a conserved hypothetical protein, in silver ion-treated *S. aureus* (Table 2).

**Table 1:** Proteins identified in *E. coli* cells treated with silver ion using MALDI-TOF MS and searching the NCBI and Swiss-Prot databases for peptide mass fingerprinting

Spot number	Protein	pI	MW	Probability^*^	Density	
					Control	Treatment
2102	Alkyl hydroperoxide reductase C22 subunit	5.0	20.86	67	577	62
3610	Phosphoglucomutase	5.5	58.60	68	989	538
4303	Fructose-1-phosphate kinase	5.4	33.97	32	1139	99
5512	Putative carbamoyl transferase	5.5	44.51	73	982	435
5610	Alpha-galactosidase	5.5	51.31	56	1157	390

*pI* isoelectric point, *MW* molecular weight

*Probability based on the Mowse scoring algorithm

**Table 2:** Proteins identified in *S. aureus* cells treated with silver ions using MALDI-TOF MS and searching the NCBI and Swiss-Prot databases for peptide mass fingerprinting

Spot number	Protein	pI	MW	Probability^*^	Density	
					Control	Treatment
2401	Carbamate kinase	5.0	34.48	83	1083	272
3511	Ornithine transcarbamoylase	5.1	37.86	51	2228	392
3620	Fumarate hydratase, class II	5.1	51.37	28	520	119
4512	Alcohol dehydrogenase	5.3	36.43	69	1698	576
6209	Conserved hypothetical protein	5.7	20.89	51	1053	361

*pI* isoelectric point, *MW* molecular weight

*Probability based on the Mowse scoring algorithm

## Discussion

Silver ions is a highly reactive moieties [11, 22] and have a high affinity for proteins [23] which probably have caused structural changes in bacteria cells. Electrostatic attraction exists between negatively charged bacterial cells and positively-charged silver ion [16] and is crucial for explaining the antibacterial effects of silver ions. The attachment of silver ions to cell proteins can significantly increase permeability, leaving the bacterial cells incapable of properly regulating transport through their plasma membrane and ultimately causing cell death. A similar effect was described by Klabunde et al. [8] when *E. coli* were treated with highly reactive metal oxide nanoparticles.

It is well known that the membrane of *E. coli* cells is predominantly constructed from tightly packed lipopolysaccharide (LPS) molecules which provide an effective barrier [4]. Amro et al. [24] have shown that attachment of a heavy metal to cell may cause the formation of irregular-shaped pits in the outer membrane and change membrane permeability, which is caused by progressive release of LPS molecules and membrane proteins. Once inside the cell, silver ions can probably bind to and denature bacterial DNA and RNA, thereby inhibiting its replication [14]. Feng et al. [15] reported the formation of electron-light regions in the cytoplasm and condensation of DNA molecules in *E. coli* and *S. aureus* that were exposed to a silver nitrate solution. Nover et al. [25] reported that a heat stimulus harmful to living cells may promote the conglomeration of some low molecular weight proteins around the nuclear region. Silver could cause a different type of shock to living cells than heat treatment; however, it is possible that some proteins that congregate around the nuclear region of silver ion-treated bacteria may protect the DNA molecules which were seen in energy-filtering transmission electron microscopy (EFTEM) results [13]. Rapid attachment and easy diffusion of silver ions into bacteria cells can cause the expansion of electron-light regions and collapse of cell walls.

It is well known that the replication of DNA is only possible when the molecules are in a relax state [26]. However, in the present study, no cell growth or multiplication was observed in bacteria that were continuously incubated with silver ions. One probable reason to this was the condensation of DNA caused by silver ions that could lower the ability of the DNA to replicate [15, 27]. It was further demonstrated that heavy metals can bind the thiol group when present inside the cell and thus inhibit the activity of various enzymes [7, 19, 28], thereby leading to cellular death [16, 29]. Silver is a type of heavy metal that can induce the deposition of proteins in the cells. Considering this, the small electron-dense granules outside the electron-light region could be deposits of silver and proteins.

The previous report suggests that the bactericidal effects of the silver ion involved interaction of silver ion with the cytoplasm in the interior of the cell using EFTEM [13, 15]. Initially, the silver ions appear to penetrate through ion channels without causing damage to the cell membranes. Once inside the cells, great affinity to bind with proteins and small size of silver ion lead it to attach and penetrate the bacterial cells that resulted in morphological distortion, particularly formation of a large gap between the cytoplasm membrane and the cell wall. Silver is a kind of heavy metal that can cause the deposition of proteins in the cells. Therefore, the entrance of silver into bacterial cells may lead to the deposition of proteins in cells.

In this study, we observed that silver ions can depress the activity of cytosolic proteins essential for glycolysis, the synthesis of amino acids, purines, pyrimidines, and nucleotides. Proteomic analysis of the silver-treated bacteria revealed decreased expression of enzymes involved in glycolysis (phosphoglucomutase, fructose-1-phosphate kinase, alpha-galactosidase, and alcohol dehydrogenase); the synthesis of amino acids, purines, pyrimidines, and nucleotides (putative carbamoyl transferase, carbamate kinase, ornithine transcarbamoylase, and fumarate hydratase class II); and detoxification (alkyl hydroperoxide reductase C22 subunit). Since the glucose pathway and tricarboxylic acid (TCA) cycle play substantial roles in the ATP production in the electron transport chain, inhibition of these enzymes by silver ions can inhibit this process which is indispensable for maintaining cell life. Inhibition of the enzymes involved in amino acid, purine, pyrimidine, and nucleotide metabolism can affect cell growth, protein synthesis, and DNA expression. Inhibition of detoxifying proteins can expose the bacterial cell to auto-oxidation because of the continuous accumulation of oxidants. Energy deficiency, inhibition of protein expression, its depressed function, and higher oxidation pressure could lead to damage and the death of silver ion-treated bacteria. These processes seem to render the cell unable to sustain membrane structures and thus cause cell disruption.

## Conclusion

The results of this study indicated that treatment with silver ions (50 μg/mL for 2 h) could kill bacteria that cause bovine mastitis (*E. coli* and *S. aureus*). Great affinity for proteins and small size enable the silver ions to attach to and penetrate the bacterial cells. Moreover, the affinity of silver ion to cellular proteins disrupts glycolysis, amino acid synthesis, nitrogen base metabolism, and detoxifying capabilities of bacterial cells. Energy deprivation, inhibition of DNA expression, and accumulation oxidants resulted in cell wall disruption and death of silver ion-treated bacteria. The results indicate that silver ion has a bactericidal capacity against bovine mastitis bacterial pathogens, which may be amenable to treatment of bovine mastitis.
